# Insights Into Macrophage Polarization and M1/M2 Balance in Diabetic Foot Ulcers

**DOI:** 10.1111/1753-0407.70205

**Published:** 2026-03-11

**Authors:** Jing Zhang, Hong Li, Yulin Dong, Zhuoyan Zhou, Yuhan Wang, Xia Chen, Yulan Cai

**Affiliations:** ^1^ Department of Endocrinology and Metabolism The Second Affiliated Hospital of Zunyi Medical University Zunyi China; ^2^ Zunyi Medical University Zunyi China; ^3^ Department of Endocrinology and Metabolism Affiliated Hospital of Zunyi Medical University Zunyi Guizhou China; ^4^ Department of Endocrinology Kweichow Moutai Hospital Renhuai Guizhou China

**Keywords:** diabetes, diabetic foot ulcers, drugs, macrophage polarization, signaling pathways

## Abstract

Macrophage polarization, encompassing classically activated (M1) and alternatively activated (M2) states, is a critical determinant of immune response in wound healing. In diabetic foot ulcers (DFUs), a persistent imbalance favoring pro‐inflammatory M1 over anti‐inflammatory M2 macrophages drives chronic inflammation and impedes tissue repair. This review delineates the central role of macrophage polarization in DFU pathogenesis and systematically summarizes the key signaling pathways that govern this process, including PI3K/AKT, PPARγ, Notch, and Toll‐like receptors (TLRs). We further synthesize these cascades into a novel hierarchical network model, identifying NF‐κB and JAK–STAT as the core regulatory hubs. Beyond mechanism, we discuss emerging therapeutic strategies—including pharmacological agents and biomaterial‐based approaches—that target macrophage polarization, positioning them as promising adjuvants to standard wound care. By integrating mechanistic insights with therapeutic potential, this review provides an updated framework for developing targeted immunomodulatory therapies to break the cycle of non‐healing in DFUs.

## Highlights

- This review elucidates the pivotal role of macrophage polarization in the pathogenesis of diabetic foot ulcers (DFUs), highlighting the contrasting functions of M1 and M2 macrophages in inflammation.
- We examine critical signaling pathways, including the NF‐κB and JAK–STAT pathways, that govern macrophage polarization in diabetic infections.
- Furthermore, we explore therapeutic strategies aimed at modulating these polarization states, offering innovative approaches for DFU treatment.
- Our findings provide valuable insights into potential interventions for improving outcomes in patients with DFUs.

## Graphical abstract

This graphical abstract illustrates pharmaceutical agents that modulate M1/M2 macrophage polarization in diabetic foot ulcers (DFUs). M1 macrophages (red) drive inflammation, whereas M2 macrophages (blue) promote tissue repair. In DFUs, persistent M1 predominance contributes to chronic non‐healing. Five key agents targeting this imbalance are depicted: insulin, AFG/GelMA hydrogel, genipin, Quercetin, and ON101 cream. These agents exert their effects through mechanisms including enhancement of M2 polarization, anti‐inflammatory activity, and modulation of critical signaling pathways (e.g., NF‐κB, JAK–STAT), thereby offering potential immunomodulatory strategies to improve DFUs healing.

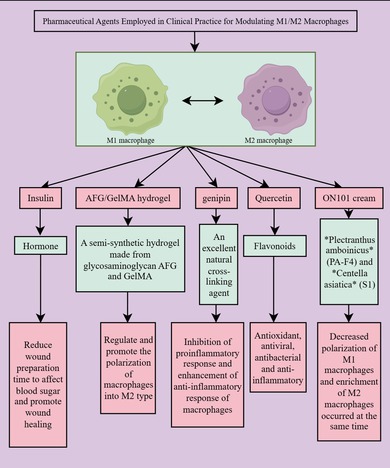

## 1 Introduction

**FIGURE 1:**
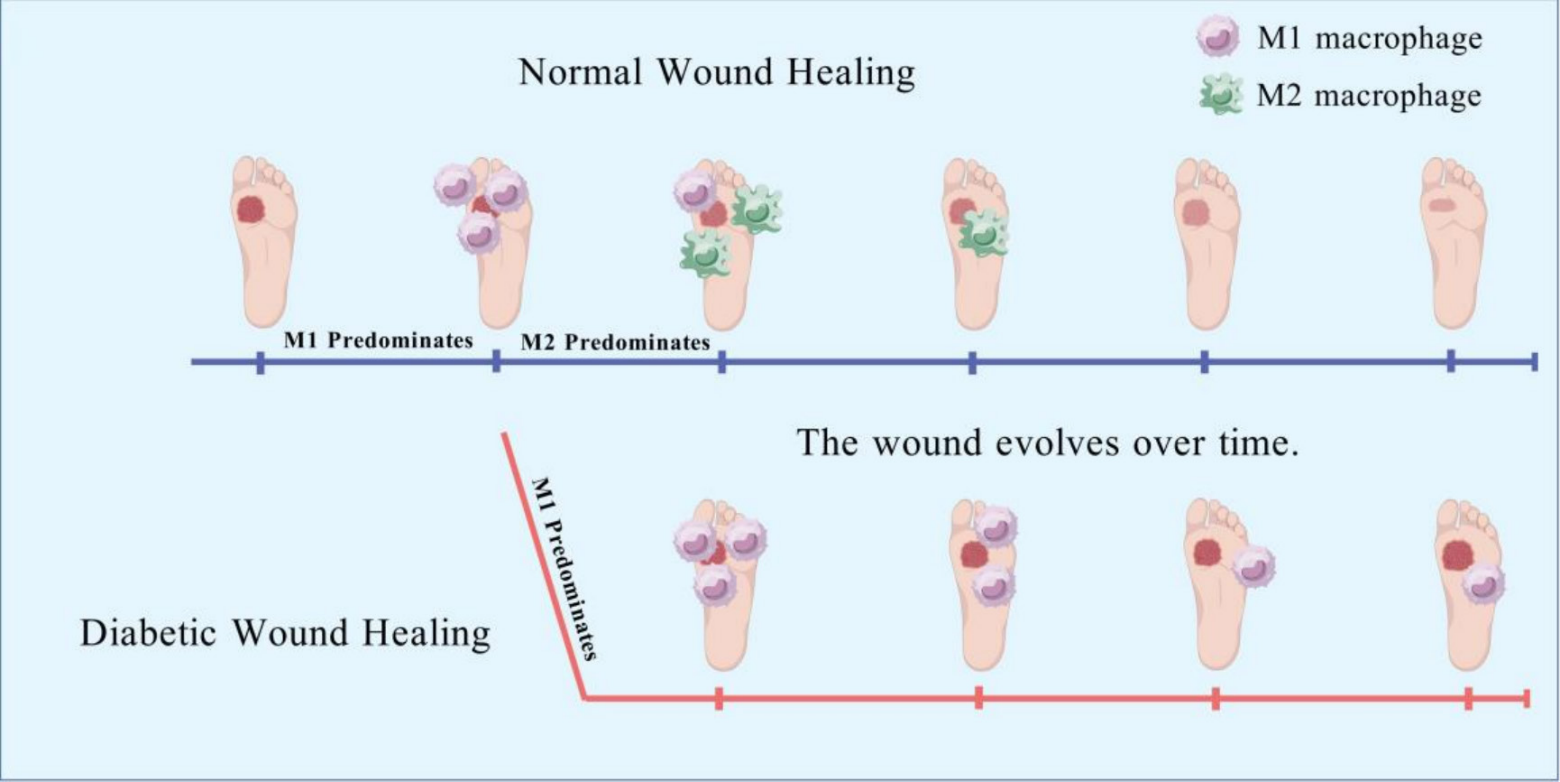
The balance of M1/M2 macrophages in diabetic foot ulcers (created with gdp.renlab.cn). By polarizing into M1 or M2 subtypes, macrophages play a pivotal role in wound healing, with M1 macrophages exhibiting pro‐inflammatory functions and M2 macrophages demonstrating anti‐inflammatory functions. The upper branch demonstrated that wound healing progressively improved under the predominance of M2 macrophages, while the lower branch exhibited gradual deterioration under the influence of M1 macrophages.

Cell proliferation, differentiation and migration are fundamental processes in wound repair, a process precisely regulated by growth factor/cytokine‐mediated signaling pathways [1, 2]. Under physiological conditions, wound healing progresses through four interconnected phases—hemostasis, inflammation, proliferation, and remodeling—requiring coordinated interactions among various cell types (including fibroblasts, immune cells, keratinocytes, and endothelial cells) and the extracellular matrix (ECM) [3, 4]. However, in chronic inflammatory conditions such as diabetes mellitus (DM), this well‐orchestrated process becomes dysregulated, leading to aberrant growth factor production, impaired cellular functions, and ultimately the development of chronic non‐healing ulcers [5, 6]. Among the multifaceted regulatory mechanisms in diabetic wound healing, macrophage plasticity represents a key component. Crucially, macrophage polarization is profoundly shaped by the local wound microenvironment. In DFUs, persistent hyperglycemia, ischemia, and infection create a pathological milieu that drives and sustains macrophage dysfunction. These immune cells dynamically shift between distinct activation states (commonly designated as M1 or M2 phenotypes) in response to microenvironmental cues, while coordinating with other cellular players in the healing process. Throughout the progression of inflammation, macrophages play dual roles, contributing to either the resolution or elimination of inflammation to prevent prolonged inflammatory responses and subsequent tissue damage. This orchestration of pro‐inflammatory and anti‐inflammatory functions is crucial for effective wound repair [7, 8] (Figure 1). When this balance is disrupted in the diabetic milieu, it perpetuates a state of chronic inflammation. Therefore, while correcting the dysregulated microenvironment is a primary goal of DFUs management—secondarily facilitating macrophage functional normalization—direct targeting of macrophage polarization itself presents a promising strategy to break the vicious cycle of chronic inflammation.

In response to harmful stimuli such as pathogens or cellular debris, macrophages trigger an inflammatory cascade, a process that necessitates signaling cascades mediated by TLRs and cytokine receptors. Subsequent to activation, macrophages undergo transcriptional and epigenetic modifications, instigating the secretion of pro‐inflammatory cytokines and chemokines, as well as facilitating cellular migration and damage clearance.

## 2 Unraveling the Intricacies of Macrophage Polarization: Insights Into Key Signaling Pathways

### 2.1 PI3K/AKT Signaling: Fine‐Tuning the Wound Healing Cascade

Protein kinase B (AKT), comprising three serine–threonine kinases—Akt1, Akt2, and Akt3—regulates a wide array of cellular functions through the AKT/PKB pathway. This pathway orchestrates processes such as wound repair, cell growth, cell survival, and modulation in response to growth factors and various extracellular stimuli [9]. The wound healing cascade is intricately modulated by the activated phosphatidylinositol 3‐kinase/AKT (PI3K/AKT) signaling pathway, orchestrating dynamic alterations in mTOR and GSK3β activity. These downstream effectors play pivotal roles in diabetic wound repair, underscoring their significance in the complex cellular responses to injury in the context of diabetes [2]. In diabetes, elevated levels of miRNA are associated with dysregulated PI3K/mTOR signaling, resulting in reduced cell proliferation and migration, alongside accelerated apoptosis [10]. The PI3K/AKT/mTOR pathway is central to signaling via various receptors, including those for insulin, pathogen‐associated molecular patterns, cytokines, adipokines, and hormones [8]. Additionally, the PDK1/AKT/GSK3β axis, another critical AKT substrate, influences key cellular processes such as polarity, motility, cell cycle, survival, and metabolism. During wound healing, PI3K/AKT‐mediated phosphorylation of GSK3β (Ser9) inhibits its activity, a mechanism that, when disrupted, contributes to the pathophysiology of diabetes [2, 11, 12]. To further elucidate the mechanisms, it is crucial to investigate the relationship between the AKT signaling pathway and macrophage polarization.

AKT acts as a pivotal signaling regulator, orchestrating a wide array of cellular functions, including cell proliferation, angiogenesis, migration, and aging [5]. The M2 macrophage phenotype is mediated by PI3K, serving as the upstream regulator of AKT [13, 14, 15]. In the context of diabetic fractures, the PI3K/AKT pathway—activated by M2‐exosomes—facilitates the conversion of M1 macrophages to M2, thereby modulating the bone immune microenvironment and accelerating the healing process [7, 16]. Activation of the PI3K/AKT and hTERT pathways has been shown to reduce inflammation and promote wound healing in DFUs by downregulating miR‐138 [17]. Additionally, hucMSCs‐HA gel has been found to accelerate DFUs healing by stimulating the MAPK and AKT pathways. Phosphorylation of AKT, p38, ERK1/2, and JNK is crucial for skin regeneration under hyperglycemic conditions [18]. Furthermore, proteomic analysis indicates that PCSK9 influences macrophage polarization by modulating EMT and PI3K/AKT signaling, with PCSK9 downregulation enhancing M1 macrophage‐mediated immunity [19].

In summary, the PI3K/AKT signaling axis serves as a crucial modulator of cellular homeostasis and wound healing, fine‐tuning macrophage phenotype. Targeting this pathway may complement broader therapeutic strategies to correct immune dysregulation and enhance tissue repair in DFUs.

### 2.2 Macrophage Polarization and PPARγ Role in DFUs Healing

In the context of DFUs healing, the genesis of wounds is closely linked to inflammation, largely modulated by key factors such as peroxisome proliferator‐activated receptors (PPARs). PPARs exert their influence by binding to specific DNA sequences, known as PPAR response elements, which play a crucial role in resolving inflammation. This is achieved by either activating the expression of pro‐catabolic genes or suppressing the expression of pro‐inflammatory genes, often through interaction with other transcriptional regulators [20, 21, 22].

Macrophage‐specific PPARγ has been shown to regulate both inflammatory signaling and lipid metabolism [23, 24, 25, 26, 27]. Under physiological conditions, the PPARγ signaling pathway facilitates wound healing by suppressing pro‐inflammatory cytokine expression and modulating macrophage phenotypes [28, 29]. Notably, a significant increase in systemic insulin resistance has been observed in mice deficient in macrophage PPARγ, accompanied by impaired maturation of anti‐inflammatory (M2) macrophages [28]. In contrast, in the diabetic wound environment, macrophage functionality is notably diminished, and PPARγ activity is impaired [30], perpetuating a pro‐inflammatory macrophage phenotype [31]. Notably, local application of PPARγ agonists has demonstrated the potential to restore macrophage homeostasis and accelerate wound closure by enhancing reparative macrophage activity [30].

Recent findings underscore the persistent activation of the NLRP3‐IL‐1β pathway in macrophages as a key driver of sustained pro‐inflammatory phenotype expression in diabetic wounds, consequently impeding the healing process [31, 32]. The prevailing consensus in current research suggests that the inhibition of peroxisome proliferator‐activated receptor (PPAR)‐γ activity in diabetic wounds is linked to the continuous production of IL‐1β. This ongoing production obstructs the critical transition of macrophage phenotypes from a pro‐inflammatory to a pro‐healing state [33].

Upon the presence of cytokines, macrophages orchestrate phenotype alterations harmonizing with changes in the surrounding microenvironment. A key player in this, PPARγ holds a pivotal role in mediating this macrophage transition from pro‐inflammatory to pro‐healing phenotype during wound healing. This modulation is further fueled by IL‐4, reported to reprogram the PPARγ pathway via STAT6, thereby magnifying PPARγ activity in macrophages [28, 34, 35]. This modulation is realized via the intricate interaction between PPARγ and signal transducer and activator of transcription 6 (STAT6) on target gene promoters of PPARγ such as FABP4 [35].

Benefitting the maturation of alternatively activated macrophages are IL‐4 and IL‐13 [20]. The binding of either IL‐4 or IL‐13 to their distinctive receptors—IL‐4Rα/IL‐2Rγc or IL‐13Rα1/IL‐4Rα—sets off a signaling cascade resulting in the cytoplasm and tyrosine phosphorylation of the transcription factor STAT6 [36]. Phosphorylated STAT6 subsequently relocates to the nucleus, triggering the expression of target genes such as marker‐like Arg1, Chi3l3, Mrc1, Mgl1, and Retnla, and alternatively activated regulators such as Pparγ, Pparδ, and PGC‐1β [37, 38]. To achieve and maintain the alternatively activated macrophage phenotype, metabolic regulators comprising PPARγ, δ, and coactivator protein PGC‐1β are obligatory [28, 39, 40, 41, 42]. Numerous studies validate the pivotal role of a shift in oxidative metabolism as an integral segment of macrophage alternative activation [34, 43]. Upon stimulation with IL‐4, macrophages undergo fatty acid uptake and oxidation alongside mitochondrial biogenesis that is transcriptionally regulated by PPARγ and coactivator protein PGC‐1β [28, 43]. These findings underscore the necessity for further research to comprehensively elucidate the underlying mechanisms of macrophage polarization and PPARγ in the healing of DFUs and to explore their potential therapeutic implications.

### 2.3 Notch Signaling Activation and Its Crosstalk With Inflammatory Pathways in Diabetic Wound Healing

The Notch signaling pathway is a highly conserved intercellular communication system composed of four membrane‐bound receptors (Notch1–4) and five canonical ligands (DLL1, DLL3, DLL4, JAG1, and JAG2). Each Notch receptor features two functional domains, namely: the Notch extracellular structural domain (NECD) and the Notch intracellular structural domain (NICD) [44]. When subjected to chronic hyperglycemic stimulation, the Notch signaling activation fosters M1 polarization of macrophages and triggers inflammatory responses. Additionally, the Notch signaling amplification enhances macrophage‐dependent inflammatory responses through NF‐κB signaling. Notably, NF‐κB is a key transcription factor that regulates pro‐inflammatory genes in M1 macrophages, including TNF‐α, IL‐1β, IL‐6, IL‐12, and cyclooxygenase‐2 [45]. The subsequent processes contribute to diabetic wound pathology by interacting with the Notch signaling and complementary pathways.

Activation of macrophages by LPS, KPB, and DLL4 leads to upregulation of Notch expression. Specifically, LPS induces the expression of the Notch ligand Jagged1 in a JNK‐dependent manner, while Notch1 activity enhances NF‐κB activation in LPS‐stimulated macrophages, thereby promoting the expression of inflammatory response‐associated genes such as TNF‐α, IL‐6, and pro‐inflammatory enzymes like iNOS [46]. Notably, IL‐6 mRNA levels are significantly elevated in Notch1‐overexpressing cells stimulated by LPS [47]. Additionally, LPS induces the expression of Notch target genes Hes1 and Hes5 in macrophages [48]. Kallikrein‐binding protein (KBP) exacerbates wound inflammation by targeting macrophages, and elevated KBP levels in DFUs have been shown to activate the Notch and NF‐κB signaling pathways, leading to M1 polarization and an increase in wound macrophages, which contributes to an excessive inflammatory response during wound healing [49]. Furthermore, studies have demonstrated elevated expression of the Notch ligand DLL4 in the skin of diabetic patients [50]. The DLL4/Notch and IL‐4/IL‐4R signaling pathways interact to influence M2 differentiation. DLL4, in conjunction with IL‐6, promotes the pro‐inflammatory M1 macrophage genotype and phenotype, as well as M1 macrophage differentiation, while concurrently inhibiting M2 macrophage differentiation by downregulating M2‐specific gene expression and increasing apoptotic cell death, thereby driving Notch‐dependent polarization shift [51].

Concurrently, Notch signaling collaborates with TLR/NF‐κB pathways to stimulate macrophages by initiating Notch1 activation through the TLR4 signaling cascade. The activation of Notch1 drives the polarization of macrophages toward the M1 phenotype via the NF‐κB transcriptional pathway [52]. Notably, the NF‐κB pathway serves as a critical convergence point for TLR and Notch signaling, working synergistically to activate Notch target genes and augment TLR‐induced cytokine production in macrophages [46]. TLR ligand stimulation upregulates Notch1 and Notch2 expression in macrophages [48]. Interestingly, overexpression of NICD1 and NICD2 has been shown to reduce TLR4‐triggered pro‐inflammatory cytokine production while enhancing anti‐inflammatory cytokine production. Furthermore, NICD1 and NICD2 inhibit TLR‐triggered ERK phosphorylation, which is crucial for Notch‐mediated suppression of TLR4‐induced inflammatory cytokine production. Notch signaling also inhibits NF‐κB transcriptional activity via the MyD88/TRAF6 and TRIF pathways, primarily through the inactivation of ERK [48].

In the initial phase of diabetic skin wound healing, impaired Notch signaling has been associated with reduced expression of inflammatory cytokines, contributing to delayed wound closure. Conversely, in the later stages, activation of Notch signaling in macrophages may exacerbate inflammatory responses. Targeted inhibition of Notch signaling could attenuate the expression of inflammatory cytokines, thereby facilitating more effective wound healing [50]. In summary, aberrant activation of Notch signaling contributes to persistent inflammation in diabetic wounds through synergistic interactions with TLR and NF‐κB pathways. Targeted modulation of this pathway holds promise as a novel immunoregulatory strategy to rebalance macrophage polarization and improve wound healing outcomes in diabetes.

### 2.4 Toll‐Like Receptors in Macrophage Polarization: Key Regulators in Diabetic Inflammation and Metabolism

TLRs function as pivotal innate immune sensors that recognize pathogen‐associated molecular patterns (PAMPs) [53]. Within innate immune myeloid cells, TLRs trigger the release of inflammatory cytokines, facilitating the generation of an adaptive antigen‐specific immune response by lymphocytes, ultimately eliminating the invading microorganism [53]. All TLRs comprise an amino‐terminal domain and a carboxyl‐terminal TIR domain, distinguished by multiple leucine‐rich repeats. The carboxyl‐terminal TIR domains interact with TiR‐containing splices. In mammals, TLRs are synthesized in the endoplasmic reticulum (ER) and subsequently transported to their ultimate cellular locations, such as the plasma membrane or endosomal membrane [54]. Upon reaching the endolysosome, TLR3, TLR7, and TLR9 initiate signaling pathways. Notably, the activation of TLR4 or other TLRs in macrophages by NF‐κB or IRF family members can prompt M1 or M2 polarization in macrophages across diverse pathological conditions [55, 56, 57].

Two junctions, MyD88 and TRIF, are known to mediate signal transduction downstream of TLR4.

Signaling through the MyD88 pathway initiates a kinase cascade involving IRAK4, TRAF6, and IKKβ, which culminates in the activation of nuclear factor κB (NF‐κB) [58]. In resting cells, NF‐κB resides in the cytoplasm as an inactive dimer bound to the inhibitor of κB (I‐κB). Upon activation, NF‐κB, a critical transcription factor in macrophage M1 polarization, orchestrates the expression of various pro‐inflammatory genes, including TNF‐α, IL1B, cyclooxygenase‐2 (COX2), IL‐6, and IL12p40 [59]. The activity of NF‐κB is tightly regulated by the IKK complex, consisting of two kinases, IKKα and IKKβ, and the regulatory protein IKKγ. Upstream signals targeting the IKK complex initially phosphorylate IKKβ, which in turn phosphorylates I‐κB, marking it for proteasomal degradation. This degradation releases the NF‐κB p65/p50 heterodimer, which translocates to the nucleus where it binds to the promoters of inflammatory genes, thereby initiating an inflammatory response [60].

Signal transduction via the TRIF pathway activates interferon response factor 3 (IRF3), which subsequently drives the expression and secretion of type I interferons, including IFNα and IFNβ. These type I interferons bind to the type I interferon receptor (IFNAR), leading to the activation of the transcription factor STAT1. This activation induces a shift in macrophages from the M1 to the M2 phenotype. The STAT‐mediated activation of macrophages is modulated by the suppressor of cytokine signaling (SOCS) family, which acts as inducible inhibitors of cytokine signaling and plays a critical role in curbing inflammatory responses [61]. Concurrently, it has been widely reported that IRF3 and IRF5 are integral to the regulation of M1 polarization and the induction of M1‐associated genes [62, 63].

Experiments have shown that TLR4 gene deletion in mice has a protective effect on adipose tissue inflammation and insulin resistance induced by high fat diet, so TLR4 plays a causal role in the metabolic changes caused by overeating and obesity. In vitro cell culture studies have shown that pro‐inflammatory cytokines produced by TLR4 signaling pathway activation have negative effects on glucose uptake and fatty acid metabolism [64, 65]. This may be an important correlation with diabetes [66, 67].

The role of TLRs in macrophage polarization is complex and multifactorial. Nonetheless, two key downstream mechanisms have been identified, underscoring TLRs and their effectors as potential therapeutic targets for modulating macrophage polarization and mitigating inflammation‐associated metabolic disturbances in diabetes.

### 2.5 JAK–STAT Signaling Is Crucial for Immune System Development and Regulation, Hematopoietic Production, Insulin Gene Expression, and Inflammation in Diabetes

The Janus kinase (JAK) family, consisting of four members—JAK1, JAK2, JAK3, and TYK2 [68, 69, 70, 71]—serves as non‐receptor intracellular tyrosine kinases that are integral to cytokine‐mediated signal transduction via the JAK–STAT pathway [72], which plays a pivotal role in regulating M1/M2 macrophage polarization. While JAK1, JAK3, and TYK2 are essential for immune system development and regulation, JAK2 is primarily involved in hematopoietic processes [73, 74, 75, 76, 77, 78, 79, 80, 81, 82]. The signal transducer and activator of transcription (STAT) family comprises seven proteins—STAT1, STAT2, STAT3, STAT4, STAT5A, STAT5B, and STAT6—each with distinct roles in cellular signaling [83, 84, 85]. There are three main types of JAK–STAT regulatory factors, namely, cytokine signal transduction inhibitors (SOCS), activated STAT protein inhibitors (PIAS) and protein tyrosine phosphatases [86, 87, 88, 89, 90]. The SOCS family, including members such as CIS, SOCS1‐7, serves as a key attenuator of JAK–STAT signaling [91, 92, 93], while the PIAS family includes PIAS1, PIAS3, PIASx, and PIASy [94, 95, 96]. The JAK/STAT signaling pathway is crucial for the activation of various cytokines and growth factors in macrophages, including IFNγ, IL‐4, GM‐CSF, and GH [97]. This pathway is also critical in pancreatic beta cells, where it mediates responses to insulin and other hormones, growth factors, and cytokines such as IFN‐γ, GH, prolactin, and erythropoietin (EPO) [97]. Notably, STAT5 has been identified as a potent inducer of insulin gene expression in vitro [98], while STAT3 is crucial for proper islet architecture development, though not essential for the function of mature islets [97]. Additionally, STAT1 is overexpressed in the islet tissues of patients with type 1 diabetes and is closely associated with the expression of HLA Class I in islet beta cells [99].

Inflammation plays a central role in the pathophysiology of diabetes [100]. The activation of the JAK2/STAT3 signaling pathway has been shown to promote M2 macrophage polarization, leading to a reduction in inflammatory cytokines such as IL‐1β, TNF‐α, and IL‐18. Concurrently, this pathway increases the levels of the anti‐inflammatory cytokine IL‐10. These changes have been associated with a significant improvement in skin lesions in a mouse model of atopic dermatitis [101]. In animal models of myocardial ischemia/reperfusion injury and psoriasis, inhibition of the JAK/STAT3 signaling pathway has been demonstrated to promote M2 macrophage polarization. This shift reduces pro‐inflammatory factors while upregulating anti‐inflammatory and pro‐fibrotic factors, resulting in a favorable attenuation of tissue inflammation and a reduction in tissue injury [102, 103]. Experimental studies have demonstrated that JAK–STAT signaling plays a critical role in TREM2‐mediated regulation of macrophage polarization. Specifically, TREM2 deficiency has been shown to promote polarization toward the M1 phenotype by upregulating STAT1 activation, while polarization toward the M2 phenotype is facilitated through upregulation of STAT3 activation [104]. The JAK/STAT signaling pathway, as a potential target in mediating macrophage M2 polarization, may offer therapeutic benefits by enhancing inflammatory wound healing, thereby promoting the repair of DFUs [105]. Furthermore, the role of STAT6 extends beyond direct gene activation; it serves as a critical facilitator for the nuclear receptor PPARγ, guiding and amplifying its transcriptional program to consolidate the M2 phenotype [106].

### 2.6 A Hierarchical Signaling Network Controlling Macrophage Polarization in Diabetic Foot Ulcers

An integration of the evidence positions the NF‐κB and JAK–STAT pathways as the central signaling backbone governing macrophage polarization. NF‐κB serves as the inflammatory “master switch,” integrating diverse danger signals from TLRs and Notch to directly drive M1 gene expression [52, 107]. Its sustained activation in diabetic wounds is a pivotal driver of M1 phenotype persistence [31, 49]. Conversely, the JAK–STAT pathway acts as the direct executor of cytokine instructions: STAT1 mediates IFN‐γ signaling to promote M1 polarization, while STAT6 and STAT3 direct M2 fate decisions in response to IL‐4/IL‐13 and IL‐10, respectively [106]. Notably, STAT6 does not act in isolation; it further guides and amplifies PPARγ transcriptional activity, thereby consolidating the M2 phenotype [35], illustrating a clear hierarchical relationship between core and regulatory pathways. In contrast, the PI3K/AKT pathway primarily functions as an important modulator, fine‐tuning the polarization process through its different isoforms and generally favoring M2 progression [8].

Consequently, therapeutic strategies targeting NF‐κB (to disrupt chronic inflammation) or JAK–STAT (to reprogram macrophage fate) may yield more fundamental impacts than focusing on auxiliary pathways. This understanding of the hierarchical signaling network provides a foundation for developing precise therapies that cooperatively target both core hubs and regulatory nodes.

## 3 Targeting Macrophage Polarization: A Therapeutic Approach in DFUs Management

As delineated in the previous sections, a spectrum of signaling pathways—with NF‐κB and JAK–STAT as central regulators—converges to drive the persistent M1‐dominant polarization observed in DFUs [106, 107]. This dysfunctional state is not merely a bystander but a key executor of the pathological healing process. The diabetic wound microenvironment, characterized by hyperglycemia, advanced glycation end products (AGEs), and ischemia, creates a persistent inflammatory milieu that impedes the critical phenotypic switch from pro‐inflammatory M1 to pro‐healing M2 macrophages [31, 108].

This sustained M1 polarization results in heightened and prolonged inflammation, hindering wound healing. Diabetic wounds exhibit delayed healing precisely when macrophages persist in the M1 phenotype and fail to transition to the M2 phenotype [109]. In the early stages of injury, M1 polarization is essential for clearing pathogens and debris [110, 111]. However, in diabetes, this response becomes dysregulated. Analysis of macrophages in diabetic wounds reveals a dysfunctional state, characterized by notably impaired efferocytosis activity and heightened production of pro‐inflammatory cytokines, which further locks them into the M1 phenotype [112]. The upregulation of molecules such as the long non‐coding RNA GAS5 in diabetic wounds is associated with increased expression of M1 macrophage marker genes, reinforcing this pathological state [113].

After the initial inflammatory phase, a timely transition to the M2 phenotype is pivotal for wound resolution, modulating inflammatory responses, and facilitating tissue remodeling [114, 115]. However, under the influence of the high‐glucose microenvironment, M1 macrophages become overactivated, leading to a pathological dominance of the M1 phenotype [16]. It is important to note that the mere abundance of M2 macrophages does not guarantee improved healing, as their function and timing are critical [116, 117]. Nevertheless, therapeutic interventions that successfully facilitate the transition from M1 to M2, such as AFG/GelMA hydrogel, have been shown to reduce inflammatory cytokines and enhance the wound microenvironment, thereby promoting the progression from chronic inflammation to proliferation and repair [118].

Consequently, targeting the molecular drivers of the impaired M1‐to‐M2 switch has emerged as a promising adjuvant therapeutic avenue to standard wound care. The following section reviews specific agents and biomaterials designed to modulate the M1/M2 balance, offering potential strategies to intervene in the chronic wound microenvironment and break the cycle of non‐healing.

## 4 Therapeutics Targeting Macrophage M1/M2 Balance in Clinical Practice

A diverse array of therapeutic strategies has emerged to modulate the M1/M2 balance, aiming to resolve chronic inflammation and promote healing in DFUs. It must be emphasized, however, that therapies directly targeting macrophage polarization are primarily considered as adjuvants to standard DFU care (e.g., debridement, off‐loading, and infection control). Their role is not to replace, but to synergize with these foundational practices by correcting the underlying immune dysregulation that impedes the healing process. The following sections discuss several promising agents, ranging from pharmacological interventions to advanced biomaterials (Table 1).

**TABLE 1:** Pharmaceutical agents employed in clinical practice for modulating M1/M2 macrophages.

Drugs	Nature	Action mechanism	Effect
Insulin	Hormone	Reduce wound preparation time to affect blood sugar and promote wound healing	It has limited applicability to relieve diabetic wound inflammation and promote wound healing
AFG/GelMA hydrogel	A semi‐synthetic hydrogel made from glycosaminoglycan AFG and GelMA	Regulate and promote the polarization of macrophages into M2 type	Biodegradation synchronously with wound healing speed, single treatment has a significant healing effect
Genipin	An excellent natural cross‐linking agent	Inhibition of proinflammatory response and enhancement of anti‐inflammatory response of macrophages	Lack of clinical trials
Quercetin	Flavonoids	Antioxidant, antiviral, antibacterial and anti‐inflammatory	Accelerate diabetic wound healing
ON101 cream	“ *Plectranthus amboinicus* ” (PA‐F4) and “ *Centella asiatica* ” (S1)	Decreased polarization of M1 macrophages and enrichment of M2 macrophages occurred at the same time	Has a better healing rate than traditional dressings

### 4.1 Timing Matters: Insulin's Pro‐Inflammatory and Pro‐Healing Effects in Wound Healing

Insulin is well‐established for its fundamental role in glucose regulation, yet it also plays a significant part in modulating inflammatory processes. The application of insulin locally is of particular interest in the context of wound healing in DFUs. Current evidence indicates that topical insulin administration may delay cellular infiltration, reduce local oxidative stress, and temper excessive inflammatory responses, collectively contributing to a reduction in wound inflammation [119, 120, 121, 122, 123]. These findings imply that insulin could represent a promising therapeutic approach for modulating inflammation and enhancing wound healing in diabetic patients.

A pivotal aspect of the wound healing process involves the polarization of macrophages, which transition between pro‐inflammatory and pro‐healing phenotypes. This dynamic shift plays a dominant role in granulation tissue formation and overall wound regulation [124, 125, 126]. In typical wound healing, granulation tissue consistently covers the ulcer surface, signifying the body's ability to counteract injury. However, in diabetes, impaired angiogenesis and reduced granulation tissue formation negatively impact the wound healing process [127, 128]. Clinical studies have confirmed that insulin application can enhance granulation tissue formation, thereby accelerating wound healing [129]. Under hyperglycemia conditions (HG), macrophage polarization leans toward a pro‐inflammatory M1 phenotype. However, insulin can propel macrophage phenotypic transformation and inhibit HG‐induced inflammatory mediator secretion [33]. The implication of the PI3K‐AKT‐Rac1 and PPAR‐γ signaling pathways in entailing insulin‐induced macrophage phenotypic transformation and anti‐inflammatory responses has been suggested, with insulin found to heighten PPARγ activity [33, 130]. Meanwhile, insulin exerts pro‐inflammatory effects during the early stages of diabetic wound healing, while promoting pro‐healing effects in the later phases [33]. The putative mechanism behind insulin's wound healing effects involves the enhancement of macrophage infiltration within the wound area, facilitating wound necrotic tissue dissolution by altering the expression of MCP‐1 on the wound surface [131, 132, 133]. Research indicates that the topical application of low‐dose insulin on diabetic wounds can induce a phenotypic transformation in macrophages. Insulin also promotes neutrophil apoptosis while enhancing macrophage phagocytosis of apoptotic neutrophils. This accelerates macrophage polarization and facilitates a phenotypic switch from pro‐inflammatory to anti‐inflammatory/pro‐healing states [33, 134]. It is interesting that heightened levels of insulin‐degrading enzymes have been found in diabetic wounds, which upon overexpression results in local insulin deficiency during the diabetic wound healing process, thereby delaying the recovery rate of the diabetic wound [134]. Local insulin injection administered to the base of the DFUs affects systemic blood sugar levels, thereby accelerating wound healing by shortening wound bed preparation time [135, 136].

The local application of insulin for treating DFUs presents a theoretically appealing approach. However, its practical application is limited by significant adverse effects. Reports have documented that local insulin injections can induce hypoglycemia, hypokalemia, and hypoaminoacidemia, thereby constraining their clinical utility [137]. Moreover, the intricate challenge of achieving precise and controlled insulin delivery to the wound bed further complicates its therapeutic application [138]. Addressing these challenges is pivotal for uncovering novel avenues in clinical treatment.

### 4.2 AFG/GelMA Hydrogel Has Many Advantages and Can be Used as a Semi‐Synthetic Adhesive for Inflammation Dissolution and Wound Repair of DFUs

The range of wound dressings currently available on the market is rapidly expanding [139]. Among these, hydrogels—hydrophilic polymers rich in polar functional groups and characterized by their high water content [140]—have emerged as promising candidates due to their resemblance to living tissue [141]. Their unique properties make hydrogels particularly suitable as wound dressings, with many being engineered as stimulus‐responsive carriers that deliver drugs or active biomolecules to not only protect but also enhance healing at the wound site [140, 142, 143]. This paper underscores that impaired wound healing, diminished angiogenesis, and dysregulated inflammatory responses are hallmark features of DFUs. The transition from inflammation to hyperplasia represents a critical step in the pathophysiology of DFUs [144, 145, 146, 147]. Currently, a growing number of hydrogels are being explored for diabetic wound repair, including Dual‐Cross‐Linked Hydrogels, ROS‐scavenging Hydrogels, Black Phosphorus Hydrogels, and AFG/GelMA Hydrogels, among others. Of particular interest is the AFG/GelMA Hydrogel, which is the focus of our discussion today [118, 148, 149, 150].

A Dual‐Cross‐Linked AFG/GelMA hydrogel is a natural adhesive found in the snail *Achatina fulica* mucus, which can be used for wound repair. The polysaccharide in this natural adhesive, known as AFG, is the main active ingredient [151]. AFG/GelMA hydrogel is a semi‐synthetic hydrogel made from glycosaminoglycan AFG and GelMA. This hydrogel has many advantages; it is easy to prepare, has a clear composition, high absorption capacity, good stability, biocompatibility and biodegradability, and more importantly it can regulate and promote the polarization of macrophages into M2 type [118]. Macrophages are the main immune cells at the wound site during skin injury, and the transformation of macrophages from a pro‐inflammatory M1 phenotype to a reparative M2 phenotype is crucial for DFUs inflammation resolution and wound repair [152]. AFG/GelMA hydrogel can significantly reduce M1 macrophage enrichment and increase M2/M1 ratio, and reverse LPS‐induced M1 polarization of macrophages in vitro [118]. AFG/GelMA hydrogel is biodegradable in sync with the wound healing rate compared to currently available viscous hydrogels, and has achieved significant healing effects with a single treatment, preventing secondary damage caused by dressing [153, 154]. AFG/GelMA also has a more stable composition than protein‐ and stem‐based hydrogels, which is important for the treatment of DFUs, as well as other clinical applications [155].

### 4.3 Genipin Cross‐Linking in Biomaterials and Genipin‐Mediated Macrophage Polarization: A Possible Treatment for DFUs

Genipin, an active component in Chinese traditional medicine, manifests potential therapeutic utilities for a broad range of ailments including metabolic disorders, inflammation, diabetes, and cancer. The crucial mechanism driving these beneficial influences remains largely unknown [156, 157, 158]. Interestingly, genipin acts as a versatile cross‐linking agent capable of effective interactions with collagen, proteins, chitosan, and gelatin, thereby facilitating their complex formation into biomaterials.

Emerging evidence highlights another aspect of genipin's therapeutic potential, specifically its function as a cross‐linker for biomaterials like collagen and chitosan, facilitating the creation of gels that accelerate wound healing [159, 160]. Studies have also demonstrated that genipin‐crosslinked collagen not only downregulates pro‐inflammatory responses but also enhances anti‐inflammatory macrophage activity [161, 162]. These genipin‐stabilized collagen biotextiles may serve as promising delivery platforms for improving the regenerative capacity of biomaterials post‐implantation [163].

In further support of the positive interaction between genipin and macrophages, in vitro studies have demonstrated genipin's capacity to promote the polarization of macrophages from the M0 to the M2 phenotype, even in the absence of other biological stimuli. Moreover, genipin maintains the M2 polarized state while the M1 subtype continues to express iNOS and IL‐1β. The evidence suggests that genipin enhances the M2 macrophage phenotype via the pSTAT6‐PPARγ pathway [163].

Utilizing genipin as a cross‐linking agent in conjunction with other biomaterials represents a novel approach for the treatment of DFUs. However, the potential of this strategy remains largely unexplored in clinical settings, highlighting the urgent need for well‐designed clinical trials to substantiate these preliminary findings. Additionally, further research is essential to elucidate the specific mechanisms by which genipin exerts its therapeutic effects in the context of DFUs.

### 4.4 Quercetin's Role in Modulating Macrophage Polarization From M1 to M2 Phenotypes for Inflammation Control and Enhanced Diabetic Wound Healing

Quercetin, a flavonoid abundantly found in fruits and vegetables, is well‐known for its antioxidant, antiviral, antibacterial, and anti‐inflammatory properties [164]. Notably, quercetin has been shown to suppress the expression of lipocalin‐2, pro‐inflammatory cytokines, and M1 polarization markers in macrophages and microglia, including IL‐1β, nitric oxide (NO), tumor necrosis factor‐alpha (TNF‐α), and interleukin‐6 (IL‐6).

Quercetin has demonstrated the ability to modulate the inflammatory response by promoting the polarization of macrophages from the pro‐inflammatory M1 phenotype to the anti‐inflammatory M2 phenotype, thereby facilitating wound healing in patients with diabetes mellitus [165]. Specifically, Quercetin effectively counteracts the lipopolysaccharide (LPS)‐induced shift toward the pro‐inflammatory M1 phenotype by inhibiting the LPS‐driven upregulation of TLR2/MyD88 and phosphorylated AMPK (p‐AMPK) [166]. Some studies have found that LPS can increase catalase activity, NO, and TNF‐α, IL‐6, and IL‐1 expression levels [167], and nuclear translocation of nuclear factors (NF‐κB) [168]. In addition, quercetin can inhibit M1 macrophage polarization through downregulation of NF‐κB and IRF5 signaling pathway activity, which causes inactivation of upstream signal TLR4/Myd88 [169]. Regulation of the PI3K/AKT/NF‐κB signaling pathway can promote M2 polarization and endogenous antioxidant expression [170]. However, while high doses of Quercetin (100 mg/kg) have been shown to exacerbate diabetes mellitus through prooxidative mechanisms, lower doses (10 mg/kg) have demonstrated antioxidant effects, potentially protecting macrophages and maintaining the oxidative/antioxidant balance. Thus, precise dosage regulation is essential to harness the therapeutic benefits of Quercetin [171]. Furthermore, research suggests promising synergistic potentials when Quercetin is combined with docosahexaenoic acid, as this combination can significantly reduce the level of pro‐inflammatory mediators [172]. This finding suggests promising new directions for advancing diabetic wound healing therapies.

### 4.5 Assessing the Efficacy of ON101 Cream in Diabetic Patients

The formulation of ON101 incorporates select fractions from “ *Plectranthus amboinicus* ” (PA‐F4) and “ *Centella asiatica* ” (S1), which have been identified for their potential therapeutic properties [173].

The therapeutic potential of ON101 in facilitating diabetic wound healing is attributed to its dual modulation of macrophage behavior, encompassing both attenuation of M1 macrophage polarization and enrichment of M2 macrophages. PA‐F4, the principal active pharmaceutical ingredient within ON101, exhibits anti‐inflammatory properties, primarily due to its capability to dampen NF‐κB‐mediated NLRP3 inflammasome activation and curtail LPS‐induced macrophage activation. Presumably, this anti‐inflammatory process encapsulates the reduction of IL‐1β, IL‐18, and IL‐6 release, vital pro‐inflammatory cytokines, from the macrophages [174]. ON101 also directly inhibits the expression of M1‐related cytokines (IL‐6, IL‐1β, and TNF‐α) and chemokines (CXCL1, CXCL9‐12, and CCL12) [175], Thus, anti‐inflammatory effects occur. In contrast, chemokines including CCL2, IL‐4, and CCL3 were upregulated after ON101 treatment, and these chemokines demonstrated M2 macrophage recruitment [176, 177, 178, 179], This is the enrichment of M2 macrophages, another mechanism by which ON101 promotes diabetic wound healing. Genome‐wide screening is critical to exploring the ways in which ON101 profoundly alters ADPC behavior [175].

A multicentre randomized clinical trial (NCT01898923) improved healing in DFUs patients based on ON101's ability to double regulate M1 and M2 macrophage subtypes. In the study, 236 eligible DFUs patients were classified as Wagner Grade 1 or 2 were randomly assigned to receive ON101 cream (*n* = 122) or absorptive dressing (*n* = 114) for up to 16 weeks. The incidence of complete healing was 74 cases (60.7%) in the ON101 group and 40 cases (35.1%) in the control group (difference, 25.6 percentage points; odds ratio, 2.84; 95% CI, 1.66–4.84; *p* < 0.001). Subgroup analyses of DFU‐related risk factors, including glycosylated hemoglobin levels ≥ 9%, ulcer area > 5 cm^2^, and DFUs duration ≥ 6 months, also indicated that ON101 had a better healing rate than traditional dressings [180]. This is crucial for the effective management of DFUs.

## 5 Conclusions

Macrophages are pivotal regulators of wound healing, dynamically interacting with stromal and immune cells to coordinate tissue repair. In diabetic foot ulcers (DFUs), this regulatory function is compromised, manifesting as impaired macrophage polarization characterized by sustained M1 dominance and a delayed transition to the M2 phenotype. This dysfunction, which acts as both a driver and a consequence of the pathological DFU microenvironment, significantly contributes to chronic inflammation and healing failure. The intricate regulation of macrophage polarization is underpinned by a complex signaling network, with the NF‐κB and JAK–STAT pathways identified as central hubs governing this process. While standard wound care (e.g., debridement, infection control, off‐loading) remains the cornerstone of DFU management by addressing the hostile wound microenvironment, targeted modulation of macrophage polarization presents a promising adjuvant immunomodulatory strategy. Recent research highlights the potential of specific drugs and therapies to correct this imbalance by targeting key signaling pathways. Although challenges in clinical translation remain, the deepened understanding of the hierarchical signaling network provides a robust foundation for developing next‐generation therapies aimed at reprogramming macrophage function and ultimately restoring the healing capacity in DFUs.

## Author Contributions

Jing Zhang and Yulan Cai conceived, supervised, wrote, reviewed, co‐created, and co‐managed the projects. Each author actively participated in the work, contributing significant intellectual content to the drafting or revision of the manuscript. They have agreed to take responsibility for their individual contributions and to ensure that any issues related to the accuracy or completeness of any part of the work are properly investigated and resolved. The final version of the manuscript to be published is subject to their approval.

## Funding

This study was supported by the: 1. National Natural Science Foundation of China (82260167; 82460167). 2. Joint Fund of Zunyi Science and Technology Bureau, No. 132, Zunshi Kehe HZ (2025). 3. Zunyi Medical University Future Clinical Excellence Physician Training Program (20231009) 4. High‐Level Talent Fund of the Second Affiliated Hospital of Zunyi Medical University (GRC‐2025‐005) 5. Guizhou Science and Technology Association Youth Science and Technology Talent Promotion Project (NO. GASTYESS202434). 6. Kweichow Moutai Hospital Science and Technology Innovation Project (MTyk2022‐42).

## Conflicts of Interest

The authors declare no conflicts of interest.
